# When Clocks Go Bad: Neurobehavioural Consequences of Disrupted Circadian Timing

**DOI:** 10.1371/journal.pgen.1000040

**Published:** 2008-05-30

**Authors:** Alun R. Barnard, Patrick M. Nolan

**Affiliations:** Neurobehavioural Genetics Group, Medical Research Council Mammalian Genetics Unit, Harwell, Oxfordshire, United Kingdom; University College London, United Kingdom

## Abstract

Progress in unravelling the cellular and molecular basis of mammalian circadian regulation over the past decade has provided us with new avenues through which we can explore central nervous system disease. Deteriorations in measurable circadian output parameters, such as sleep/wake deficits and dysregulation of circulating hormone levels, are common features of most central nervous system disorders. At the core of the mammalian circadian system is a complex of molecular oscillations within the hypothalamic suprachiasmatic nucleus. These oscillations are modifiable by afferent signals from the environment, and integrated signals are subsequently conveyed to remote central neural circuits where specific output rhythms are regulated. Mutations in circadian genes in mice can disturb both molecular oscillations and measurable output rhythms. Moreover, systematic analysis of these mutants indicates that they can express an array of abnormal behavioural phenotypes that are intermediate signatures of central nervous system disorders. Furthermore, the response of these mutants to psychoactive drugs suggests that clock genes can modify a number of the brain’s critical neurotransmitter systems. This evidence has led to promising investigations into clock gene polymorphisms in psychiatric disease. Preliminary indications favour the systematic investigation of the contribution of circadian genes to central nervous system disease.

## Introduction

The correct functioning of the endogenous circadian clock enables organisms to anticipate daily environmental changes and temporally modify behavioural and physiological functions appropriately. All organisms maintain a large number of physiological variables (sleep-wake cycle, locomotor activity, temperature regulation, water/food intake, and levels of circulating hormones) under control of the circadian clock. There are well-known consequences of disrupted circadian function outside of the brain; metabolism, reproduction, and even longevity can be adversely affected when the means of determining time of day are altered at a molecular level (for review see [1]). Perhaps less has been said regarding the brain and the idea that altered clock function may contribute to neurological, behavioural, and psychiatric deterioration. Here, we explore the contribution of this complex circadian system to brain function in physiological and diseased states. Following a description of the molecular and neural basis of mammalian circadian oscillations and their relationship to brain function, we consider the wider behavioural effects of mutations and ablations of circadian system genes in mice. Furthermore, we explore the persistent observation of rhythm misregulation in psychiatric and neurological disorders and in their related mouse models. Finally, in considering the contribution of circadian regulation to disease symptomatology, we examine the association between clock gene polymorphisms and behavioural traits and disorders in humans. Although sparse, these reports suggest that clock gene misregulation can contribute to the severity of a broad spectrum of central nervous system (CNS) diseases.

## Molecular and Neural Basis of Mammalian Circadian Oscillations

**Figure 1:**
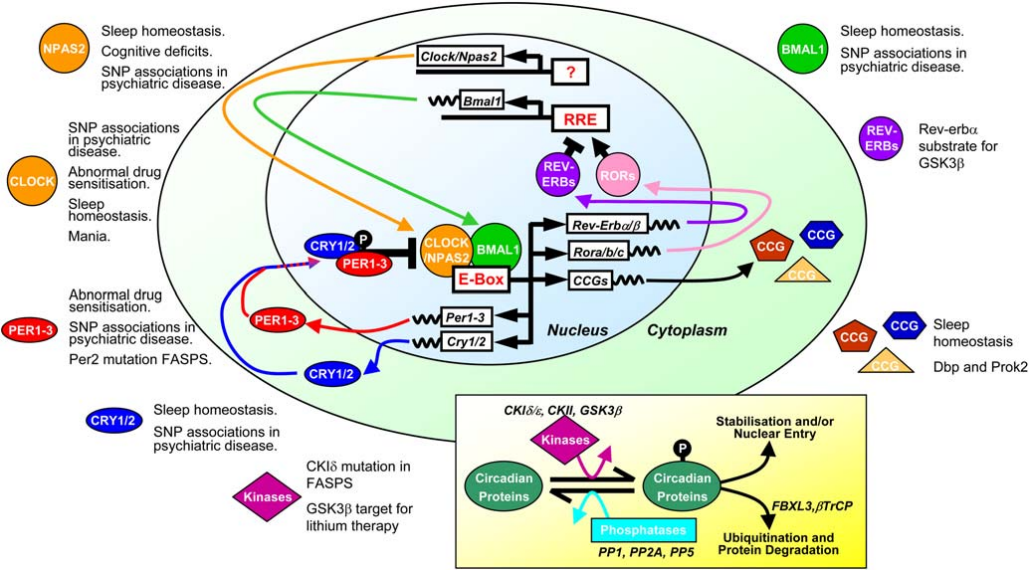
The Mammalian Molecular Circadian Oscillator. The molecular circadian oscillator incorporates numerous transcriptional and posttranslational elements. Disruptions in many of the individual circadian elements in mice can lead to behavioural disturbances that mirror endophenotypes in human neurological and psychiatric disorders. Moreover, some studies have established circadian gene polymorphisms in psychiatric conditions and mutations in behavioural syndromes (see text for details). The central component of the figure depicts the core mammalian circadian feedback loop. CLOCK(or NPAS2):BMAL1 heterodimers drive the transcription of multiple genes (*Cry1/2*, *Per1-3*, *Rev-Erba/b*, *Rora/b/c*, multiple CCGs) through E-box elements. Nuclear accumulation of CRY and PER proteins can inhibit CLOCK:BMAL1-mediated transcription by directly interacting with the complex (black bar–ended arrow). As PER and CRY levels fall, the negative repression is lifted and CLOCK:BMAL1-driven transcription re-occurs. In an additional stabilising loop, REV-ERB and ROR proteins co-regulate the transcription of *Bmal1* by competing for RREs in its promoter sequence. Rhythmic output of the clock is achieved through E-box elements in CCG which can impact a range of cell processes and physiology. The stability and subcellular localisation of circadian proteins is highly regulated by kinases and phosphatases (inset box). Although not entirely understood, the phosphorylation state of circadian proteins can affect their cellular localisation and/or stability. Mutations affecting the stability of Per proteins can accelerate the molecular clock in humans, leading to the inherited syndrome familial advanced sleep phase syndrome (FASPS).

The suprachiasmatic nucleus (SCN) of the hypothalamus is the centre for the circadian system in the mammalian brain [2]. Through its neuronal and humoral outputs, the SCN can orchestrate a number of physiological and behavioural rhythms throughout the body. Perhaps surprisingly, these circadian rhythms are not derived from the multicellular network properties of the SCN but are in fact generated at a cell autonomous level. The endogenous clock is fundamentally encoded, at the molecular level, by interlocking autoregulatory transcriptional and translational feedback loops within every cell (see [1],[3] for reviews and Figure 1 for description). The same coordinated molecular circadian rhythms have been identified in a number of cell types, tissues, and organs throughout the body. This observation also applies to a wide range of CNS regions, including the olfactory bulb, pituitary, pineal gland, arcuate nucleus, and retina [4]–[11]. Importantly, it has been established that these rhythms are not externally driven by the output of the SCN, and the time or phase at which particular gene products peak is often region-specific [4],[5].

**Figure 2:**
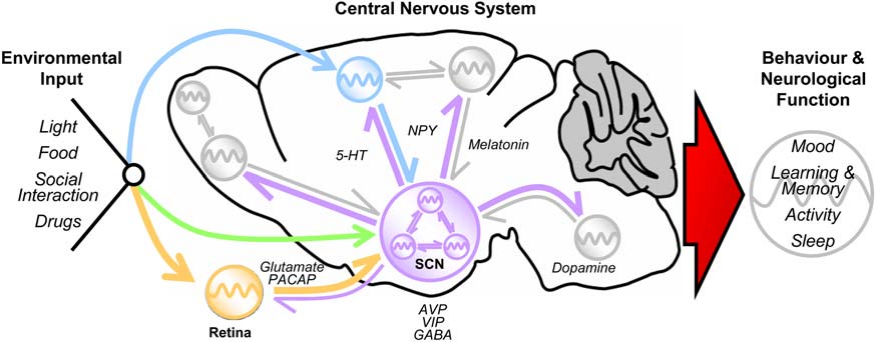
The Circadian System and the Mammalian Brain. The SCN acts as a “master” clock, sending neuronal and humoral output to a number of regions in the CNS. The SCN can passively drive rhythmicity in these regions, or nuclei may themselves have their own automomous clocks. Within the SCN, every cell is a potentially independent oscillator unit. Extensive intra-SCN signalling is used to synchronise the rhythms of multiple cells. Entrainment is a process whereby environmental stimuli can alter the timing or phase of central rhythms so that they are coincidental with the prevailing daily cycle (far left). Time cues reach the SCN via several pathways involving intermediate brain areas. Some of these relay nuclei may contain their own oscillators and/or have rhythms in their function (blue and orange arrows and oscillators). Others may not have rhythmic function (green arrow). Extra-SCN oscillators may directly utilise time cues (blue and orange oscillators) or may rely solely on the SCN to synchronise/entrain rhythms (lilac arrows to grey oscillators). These oscillators may also provide feedback which impacts on the operation of the SCN (grey return arrows). In addition, extra-SCN oscillators may drive/influence rhythmicity in other brain areas (smaller grey oscillator) and communicate with each other independently from the SCN (reciprocal grey arrows). Within the CNS, some neurotransmitters/neuromodulators involved in signalling are indicated. The overall output of this complex system (large red arrow) creates the daily rhythmicity seen in a range of neurological functions and behaviours (far right). There is a wider communication with clock/oscillator units throughout the entire body. However, these so-called “peripheral” oscillators and their actions are not shown here.

Aside from generating robust rhythms, the circadian system must be sensitive to environmental cues and be able to adjust its phase to synchronise with the prevailing day/night rhythm, a process known as entrainment. In mammals, the signalling of environmental light exposure, carried by dedicated retinal afferents, is the most important entrainment cue of the SCN clock [12]. However, other cues such as food availability, novel environment, or social interaction may act as timekeeping cues (Figure 2). These signals reach the SCN clock through disparate neurotransmitter/neuromodulator and hormonal pathways and can reset the phase of expression within the autoregulatory feedback loops [13]–[16]. Non-SCN clocks can be directly sensitive to external timekeeping cues; the most obvious example is the retina [10],[11], although there is evidence that additional, extra-SCN oscillators receive relevant environmental phase-setting information directly [17]–[19]. However, as they also receive strong entrainment signals from the SCN [6],[8], it is a process of bidirectional communication and feedback that ultimately establishes the entrainment and phasing of molecular clocks throughout the brain and body. A final level of complexity and fine-tuning in molecular rhythms may also be derived from local crosstalk between clock tissues and nuclei (reviewed in [8]).

For molecular circadian rhythms to have an impact on physiology and behaviour, they must be converted into functionally relevant outputs. This is achieved by complex spatiotemporal regulation of gene expression that impacts on cell/tissue-specific function. The orchestrated expression of a number of so-called clock-controlled genes (CCGs) then dictates the temporal regulation of clock-output/functional rhythms in brain regions/systems and peripheral tissues. These may take the form of altered hormone release, changes in action potential firing rate, modified rates of cellular metabolism, etc. [3].

All of the above indicates that the generation of daily rhythms in behavioural and physiological functions, together with the ability to integrate a range of environmental inputs, relies on a complex circadian system. And evidently this system relies on the action of many genes. Moreover, novel genetic factors that influence circadian parameters continue to be identified in mutagenesis screens [20],[21], in QTL analyses [22],[23], and in protein interaction studies [24].

## Neurological and Behavioural Phenotypes in Mouse Clock Mutants

Molecular-genetic modifications of the circadian system have a wide range of effects depending on the particular gene affected and also the nature of the mutation (Figure 1). Investigation of mouse clock mutants has focussed primarily on circadian behaviour by assessing overt activity rhythms and molecular rhythms in the SCN. The effects of circadian gene manipulation can range from the profound (complete loss of behavioural and molecular rhythmicity in constant conditions [25]–[27]) to subtle effects (e.g., altered phasing of behavioural/molecular rhythms [28]). However, a more systematic behavioural characterisation of mouse clock mutants could potentially uncover novel behavioural phenotypes associated with clock genes. Given the role of the SCN molecular oscillator in synchronising and entraining neural rhythms in discrete brain centres, clock mutants might affect CNS function through a primary deficit in SCN oscillator function, a deficit in discrete brain region oscillator function, or as a secondary consequence of specific neural rhythm dysfunction.

Disturbances in sleep parameters are associated with a spectrum of neurological and psychiatric disorders. Sleep patterns are affected not only by circadian timing mechanisms but also by independent homeostatic mechanisms that determine the amount of sleep required [29]. Analysis of sleep parameters predicts that mechanisms other than the regulation of sleep-wake timing are affected in clock mutants, as sleep anomalies in clock mutants do not always mirror the behavioural rhythm anomalies. Mutations in *Clock* (MGI:1861634), *Bmal1* (MGI:2180361), and *Cry1* (MGI:1270841)*/Cry2 *(MGI:2181206) [30]–[32] result in alterations in sleep time, sleep fragmentation, and atypical responses following sleep deprivation. Similarly, a number of other clock-related genes, including *Npas2* (MGI:109232), *Dbp* (MGI:94866), and *Prok2* (MGI:1354178) affect elements of sleep homeostasis [33]–[35]. However, further evidence exists for the functional segregation of circadian and homeostatic regulation of sleep, as *Per1* (MGI:2182834)*/Per2* (MGI:1934020) double mutants show no effects on sleep homeostatic mechanisms, although robust rhythms of sleep and wakefulness are lost in constant conditions [36],[37]. Thus, upon consideration of this data, effects of clock mutations on sleep homeostasis could be related to pleiotropy or brain region specificity of particular clock genes.

The process of memory formation in clock mutant mice has received limited attention. *Npas2* can deputise for *Clock* in maintaining molecular rhythms in the SCN and is in fact the normal binding partner for *Bmal1* in forebrain clocks [9],[38]. Mutants lacking *Npas2* show deficits in the acquisition of cued and contextual fear-conditioning paradigms [39]. It is not feasible that the deficit in *Npas2* null mice is secondary to major, centralised circadian disruption, as these mice show only a subtle activity phenotype. A pleiotropic, non-clock effect of *Npas2* in memory formation is one possible explanation. Alternatively, the loss of *Npas2* may abrogate particular extra-SCN brain clock(s) that regulate memory formation. Evidence for the participation of other clock genes in memory regulation is sparse. Although no specific memory deficits have been found in *Per1/Per2* mutant mice [40], *Drosophila Per* mutants (FBgn0003068) are defective in long-term memory formation [41]. In the same study, no memory-associated deficits were found in *timeless* (FBgn0014396), *clock* (FBgn0023076), and *cycle* (FBgn0023094) mutants, indicating that an association between clock genes and memory regulation is not universal and a degree of functional segregation exists within the system.

There is increasing evidence that a dysfunctional circadian system could be a primary cause of altered emotional behaviour. The majority of this evidence in mice comes from studies on the ENU-induced mutation *Clock*. Homozygous mutants have a spectrum of behavioural abnormalities including low anxiety, mania, and hyperactivity [42],[43]. These behavioural abnormalities are perhaps explicable by the direct effects of the *Clock* mutation on core molecular circadian oscillations or are secondary to an alteration in the dynamics of communication between CNS clocks dysregulated by the altered function of the core oscillator. In support of the second possibility, behavioural disturbances in homozygotes are associated with increased dopaminergic activity [42],[43]. Further work strengthens the link between the circadian system and mood regulation. Dysregulation of either NPY or VIP, important neurotransmitters for signalling to and within the SCN, can lead to a number of behavioural disturbances, including alterations in anxiety-like behaviour and aggression [44],[45].

The observations that the mood-stabilising agent, lithium, can reverse behavioural disturbances in *Clock* mutant mice [43] adds to an increasing body of evidence that lithium’s therapeutic effects may be partially mediated through its effects on the circadian system. Lithium is a potent inhibitor of glycogen synthase kinase 3β (GSK3β, MGI:1861437). Mutations in the *Drosophila* homologue, *shaggy* (FBgn0003371), can lengthen the circadian period [46]. In mammalian systems, GSK3β can mediate its circadian effects through promoting the nuclear expression and stability of a number of clock proteins [47],[48]. Moreover, in cultured mammalian cells lithium treatment leads to the rapid proteasomal degradation of the clock protein Rev-erbα (MGI:2444210) and subsequent activation of the clock gene *Bmal1* (MGI:1096381) [48].

Additional evidence for behavioural disturbances in clock gene mutants is supported by the fact that they appear to affect the sensitisation to, and preference for, drugs of abuse. This was first evidenced in a number of *Drosophila* mutants including *period*, *clock*, *cycle*, and *doubletime* (FBgn0002413) [49], where sensitisation to the effects of cocaine is eliminated. Similar effects were identified in some mouse mutants, although some had opposite effects. For example, mouse *Per1* mutants show no behavioural sensitisation to cocaine, and, in a conditioned place preference task, show no preference to cocaine [50]. *Per2* mutants have a hypersensitised response to cocaine, and the place preference response is strong [50]. Mice carrying the ENU-induced mutation *Clock* also show a hypersensitive response to cocaine and increased place preference [51]. These authors suggest that the increased expression and phosphorylation of tyrosine hydroxylase in mutants may affect dopaminergic function within the brain’s reward circuits. Effects of clock mutants on the brain’s neurochemical systems are not limited to the dopaminergic system. Expression of the glutamate transporter *Eaat1* (MGI:99917) is reduced in mouse *Per2* mutants, leading to decreased uptake of glutamate by astrocytes and increased extracellular glutamate levels [52]. Moreover, increases in alcohol consumption in *Per2* mutant mice were reversed by agents believed to act through reducing abnormally high glutamatergic activity. Overall, the interactions between circadian clocks and addiction mechanisms seem to be complex. The possibility of pleiotropy in clock genes cannot be discounted, although these effects could be dissected using conditional mutants. Nevertheless, there is an increasing body of work detailing the interaction between the circadian system and brain neurotransmitter systems. As described above, clock gene expression can be modulated by a number of afferent neurotransmitter effects, and, conversely, the functions of efferent neurotransmitter systems/brain nuclei are modifiable by altered clock gene expression [42],[43],[52].

## Altered Circadian Parameters in CNS Disorders

Undoubtedly, circadian parameters are consistently disrupted in a spectrum of CNS disorders (Table 1). In many cases, these disruptions may be secondary to compromised neural circuitry where brain regions regulating output rhythms are disturbed. Nevertheless, it has been difficult to establish whether circadian system disturbances can contribute to CNS disorders or whether they are merely symptomatic of the disease process. In all likelihood, disruption of circadian oscillators can at least modify disease severity, whereas, in some instances, they may play a more primary role in the aetiology of the disease.

**Table 1:** Rhythm/Sleep Endophenotypes in Human CNS Disease and in Mouse Models.

Human Disease or Condition	Disturbed Rhythm/Sleep Endophenotype	Relevant Phenotypes in Mouse Models
Familial advanced sleep phase syndrome (FASPS)	Early sleep and wake times, shortened circadian rhythms [112].	Mice expressing human mis-sense mutations in *Per2* or *CKIδ* have advanced phase of activity in a light-dark schedule and a shortened activity rhythm [92],[102].
Delayed sleep phase syndrome (DSPS)	Extreme evening preference, delayed phase of activity, sleep, core body temperature, and melatonin [113].	No model.
Seasonal affective disorder (SAD)	Depressive symptoms occur during shorter winter days [88]–[90].	No model.
Mood disorders (unipolar depression) and psychoses (schizophrenia, bipolar)	Depression. Increased sleep latency, impaired sleep continuity, phase advance in endogenous circadian system relative to sleep schedule, phase advances in growth hormone, plasma melatonin, increased plasma cortisol at night. All major affective disorders include circadian phase disturbances in sleep, activity, temperature, and hormone levels (for reviews see [84]–[86]).	No accurate mouse model. Mutants in serotonergic and dopaminergic systems show disturbances in circadian phase and/or sleep parameters [94]–[97]. *Clock* mutant has low anxiety, mania, and hyperactivity [42],[43]. Cognitive disturbances in *Npas2* mutant [39]. Abnormal sensitisation to drugs of abuse in *Clock* and *Per* mutants [50]–[52].
Autism spectrum disorders (ASD)	Longer sleep latency and greater sleep fragmentation. Abnormalities in circadian rhythm and mean concentration of plasma melatonin [81].	Mice expressing a conditional deletion of *Pten* have a significantly longer free-running period [83].
Down syndrome	Reduced sleep maintenance, sleep fragmentation, reduction in percent REM sleep, sleep apnea [73]–[75].	Ts65Dn mouse mutant shows increased activity in the light phase, a reduction in rhythm amplitude, and a 4-h advance in the phase of activity [76],[77].
Smith-Magenis syndrome	Inverted rhythm of melatonin secretion [69],[71]. Advanced sleep/wake phase. Nighttime wakening, daytime sleepiness. Reduced total and NREM sleep [70].	Heterozygous deletion mutant mice have a hypoactive phenotype and a significantly shorter circadian period [72].
Prader-Willi syndrome	Sleep apnea, sleep-related and behavioural disturbances including daytime napping and excessive daytime sleepiness [75],[78],[79].	Mice deficient for mage-like 2 gene (Magel2) have a reduced circadian activity amplitude with increased daytime activity [80].
Parkinson disease (PD)	Sleep fragmentation, sleep apnea, REM sleep behaviour disorder, excessive daytime sleepiness [53].	No recorded circadian or sleep disturbances in genetic mouse models [114].
Huntington disease (HD)	Nocturnal awakening and progressive disintegration of daily activity rhythms [55].	R6/2 mouse transgenic line has increased daytime and reduced nocturnal activity. Progresses to a complete disintegration of diurnal and circadian activity rhythms [55].
Alzheimer disease (AD)	Fragmented sleep, increased nocturnal activity, and reduced daytime activity. Delayed phase in peak of daily activity [57].	Alterations in sleep regulation and timing in Tg2576 [62] and PDAPP mice [61]. Tg2576 mice also have a significantly longer circadian period [62]. Both TgCRND8 and APP23 mice show changes in daily activity profiles potentially analogous to those seen in AD patients [63],[64].
Aging	Sleep disturbances due to earlier wake time and reduced sleep consolidation. Partially attributed to age-related reduction in amplitude and advance in phase of circadian rhythms [56],[58].	Aging lengthens the period and reduces the amplitude of circadian activity rhythms. The onset of daily activity is significantly delayed and the variability of onset is increased [67].
Prion diseases	Severe sleep abnormalities, progressive loss of circadian rest-activity, and melatonin rhythms [54],[59],[60].	Increased sleep fragmentation and significantly longer circadian period in activity in prion protein null mutants [65].

In neurodegenerative disease and aging (Table 1), disturbances in sleep mechanisms and circadian disturbances in phase and amplitude of activity and temperature regulation are common symptomatic features secondary to a deterioration in brain circuitry [53]–[60]. Circadian activity disturbances have been described in a number of transgenic mouse models of Alzheimer Disease (AD, OMIM; #104300), and in some cases these precede the onset of typical AD pathologies [61]–[64]. The neurodegenerative prion diseases also express a spectrum of sleep and circadian rhythm disturbances [54],[59],[60]. Interestingly, these disturbances are mirrored in mice that are null for the prion protein [65]. In the Huntington R6/2 mouse transgenic line, disintegration of activity rhythms mimic those seen in Huntington patients (OMIM; +143100), and these behavioural disturbances are accompanied by altered clock gene rhythms in the SCN, motor cortex, and striatum [55]. Similarly, aging alters activity onsets, lengthens circadian period, reduces circadian amplitude, and alters SCN electrophysiology in mice [66],[67]. Age-related circadian changes may also be symptomatic of brain circuitry deterioration as, for example, comparable effects of aging are seen in wild-type and *Clock* mutant mice [68].

Circadian disturbances are more likely to contribute to the aetiology of a number of syndromic disorders (Table 1). These disturbances are likely to be due to deficits in specific oscillating neural and molecular circuits that are regulated by the clock. Smith-Magenis syndrome (SMS, OMIM #182290), associated with heterozygous deletions on Chromosome 17, is characterised by significant sleep disturbances with a reversed secretion rhythm of melatonin [69]–[71]. Mice carrying an engineered heterozygous deletion of the SMS syntenic region (Del(11Cops3-Zfp179)1Jrl, MGI:3521985) have a hypoactive phenotype and an abnormal circadian period [72]. Sleep disturbances are also features of Down syndrome (OMIM #190685) patients [73]–[75], and the Ts65Dn mouse mutant expresses a number of abnormal circadian parameters including increased activity in the light phase, a reduction in rhythm amplitude, and a four-hour advance in the phase of activity [76],[77]. Patients with Prader-Willi syndrome (PWS, OMIM #176270) also exhibit a spectrum of sleep-related and behavioural disturbances [75],[78],[79]. Mice deficient for the mage-like 2 gene (*Magel2*), an SCN-enriched transcript within the PWS deletion region, have a reduced circadian activity amplitude with increased daytime activity relative to controls (MGI:3760092) [80].

Other behavioural disorders with circadian and sleep-related disturbances include autism spectrum disorders (ASD) (OMIM %209850) [81]). Behavioural disturbances in ASD may arise in part from an inability of an individual’s circadian oscillator to entrain to environmental and social cues. One specific correlate of ASD is a low level of melatonin, and one of the enzymes critical in the synthesis of melatonin, acetylserotonin-O-methyltransferase (*ASMT*, OMIM *300015), is implicated as a susceptibility gene for ASD [82]. Mutations in the phosphatase and tensin homologue on Chromosome ten (*PTEN*) have been reported in autistic individuals with macrocephaly. In mice carrying a conditional deletion of *Pten* (MGI:2182005), abnormal phenotypes include macrocephaly, increased susceptibility to seizures, social interaction deficits, anxiety, and a significantly longer free-running period [83].

The contribution of the circadian regulatory system, arising from conflicts between internal biological clocks and environmental (solar) and social clocks, is evident in affective disorders. All major affective disorders (such as unipolar depression, OMIM #608516; bipolar disorder, and schizophrenia, OMIM #181500) include circadian phase disturbances in sleep, activity, temperature, and hormone levels (for reviews see [84]–[86]). Moreover, there is evidence that if rhythms can be altered/stabilised using relevant therapies, improvements in the primary symptoms can occur. For example, in some instances sleep deprivation has an antidepressant effect in patients [87]. Conversely, many disorders with a primary anomaly in the circadian system are associated with depressed mood. Seasonal affective disorder (SAD; OMIM #608516) is a common condition where depressive symptoms occur during shorter winter days [88]–[90]. Two inherited sleep phase disorders, familial advanced sleep phase syndrome (FASPS; OMIM #604348) and delayed sleep phase syndrome (DSPS), are both associated with abnormal affective states [91],[92]. Furthermore, individuals with a behavioural preference for “eveningness” have a greater tendency to develop depression [93]. Because of the difficulties in classifying these diseases/conditions in humans and because of their multigenic nature, it is difficult to study their aetiology in mouse models. However, although no mouse mutants have been developed to model complete affective disorders, a number of mutants in serotonergic and dopaminergic systems displaying features of these disorders also show disturbances in circadian phase and/or sleep parameters [94]–[97].

## Polymorphisms in Human Clock Genes Alter More Than Rhythms

The contribution of circadian gene dysregulation to CNS disorders has only recently been explored through the analysis of clock gene polymorphisms. Circadian gene polymorphisms may simply underlie a morning or evening preference in human populations, but extremes in these behaviours may also underlie the more complex phenotypes associated with psychiatric disorders. A quantitative scoring system for diurnal preference, the Horne-Ostberg questionnaire [98], has been used as the basis for association studies, and these have been carried out either in random populations or in families inheriting FASPS or DSPS. Many studies implicate altered dynamics in clock protein phosphorylation as a critical factor (see Figure 1). For example, the shorter 4-repeat allele of a 54-bp coding-region polymorphism in the *PER3* (OMIM *603427) gene is associated with extreme evening preference and DSPS [99]. Moreover, in comparisons between short- and long-repeat patients, slow wave sleep was greater in 5-repeat individuals, and deficits in cognitive performance following sleep loss were greater [100]. This 54-bp sequence is in a region of *PER3* encoding a putative phosphorylation domain. The significance of these observations has been highlighted by the identification of two distinct mutations in familial ASPS pedigrees. The first [101], a mis-sense mutation in *PER2* (OMIM *603426), is within the binding domain for casein kinase I epsilon (CKIε; OMIM *600863) and results in hypophosphorylated PER2 in vitro. Transgenic mice expressing this mutation had an advanced phase of activity in a light–dark schedule and a short free-running period of activity [102]. A second mis-sense mutation was found in *CKIδ* (OMIM *600864) itself [92], the mutation resulting in reduced enzymatic activity in vitro. Again, the phenotype in a mouse transgenic line mimicked that of the human individuals.

Conflicts between internal biological clocks and environmental (solar) and social clocks should be evident in individuals with extreme diurnal preferences, and this could have the potential to disrupt homeostatic processes and behaviours affected by them. In fact, Xu et al. [92] observed that four out of five patients with FASPS showed either evidence of the clinical features of, or a history of, depression. Observations such as this and the hypothesis that certain genetic susceptibility factors are shared across the psychosis spectrum [103] have led several groups to investigate clock gene associations in a number of mood disorders and psychoses. The most evident example is in SAD. Partonen et al. [104] found that SAD was associated with SNP variants in *PER2*, *BMAL1* (OMIM *602550), and *NPAS2* (OMIM *603347) with additive effects from combined risk genotypes. An earlier study had also identified significant associations between diurnal preference and SAD with a significant effect of NPAS2 471 Leu on disease susceptibility [105].

Investigations into clock gene associations with other affective disorders are in their infancy. Although limited, there are a number of indications warranting further investigation into associations with bipolar disorder, schizoaffective disorder, schizophrenia, and autism [106]–[111]. Variants of *PER1* (OMIM *602260), *PER3*, *NPAS2*, *BMAL1*, *CLOCK* (OMIM *601851), and *TIM* (OMIM *603887) all showed specific associations with a number of disorders. The promise of these pilot studies should encourage more systematic studies in investigating the contribution of clock gene variants to the onset and severity of CNS disorders.

## Conclusions

Biological rhythms are undoubtedly disrupted in a spectrum of CNS disorders. Establishing the contribution of clock genes to cause and effect in these disorders has proven difficult. Nevertheless, these investigations continue to be helped through the systematic characterisation of behavioural phenotypes in mouse circadian mutants, the identification of new genetic factors that contribute to circadian and behavioural function, and the investigations of clock gene polymorphisms in all CNS disease areas. Continued investigation into these three areas should lead to new insights into the causes and progression of neurological and psychiatric disorders.
